# Preservatives in Food

**Published:** 1906-12-29

**Authors:** 


### PRESERVATIVES IN FOOD.

A Conference was held at the Institute of Hygiene on the 29th ult. on "Preservatives in Food: Their Use and Abuse." Professor Tunnicliffe, M.D., who occupied the chair, opened the conference by a sketch of the development of the use of preservatives in food. The employment of these is by no means a new thing, for it includes such common substances as salt, sugar, and alcohol; but for convenience of discussion these were not dealt with, and the so-called chemical preservatives alone were spoken of. Dr. Tunnicliffe gave expression to carefully moderate views on the subject, and, while he thought it was a legitimate matter for discussion whether a weak solution of boracic acid was not less injurious to health than a strong solution of salt, he by no means ignored the dangers to which the use of preservatives may give rise. He pointed out that preservatives may be used to cover bad workmanship in the preparing of the foods, and that they are more harmful in some foods than in others. The medical profession was quite alive to the possible dangers of the use of these chemicals, but we wanted to know more of these substances, not as drugs but as articles of domestic use. It was possible that chemicals in food might be the cause of secret and mysterious illnesses, but what was wanted was facts. On the whole, however, Dr. Tunnicliffe seemed to think that preservatives were not so harmful as many people thoughtlessly assumed, and he pointed out that "the kipper, which contains chemical substances in dreadful variety and atrocious complexity," is not only a popular but in most cases a harmless article of diet.

Professor Tunnicliffe was followed by Dr. Otto Hegner, who moved the first resolution :—

"That it is essential, for the protection of the public, that definite regulations be made as to the conditions under which the use of preservatives in foodstuffs shall be permitted, such regulations to deal with (a) the articles in which preservatives may or may not be used, (b) the nature and maximum of anything so permitted as a preservative, and (c) the conditions under which articles containing a preservative shall be sold."

In speaking to the resolution, Dr. Hegner dwelt on the fact that in England alone of all civilised countries there was no regulation as to the use of preservatives in food. The seriousness of this reproach, however, was to some extent discounted by the remark of a subsequent speaker, Mr. Giles, secretary of the Grocers' Federation, who said that the regulations in some European countries had been made more in the interests of political parties than in those of the consumers, and who stated that in Denmark, where the use of preservatives is forbidden, there are more deaths from kidney trouble than in England. Dr. Hegner did not, however, wish to put an end to the use of preservatives, and admitted that there was not much obvious danger to health in the use of preserved articles of diet. Indeed, in a country like England, which imported so much of its food, it was impossible to avoid them. Although imported meat was brought to England in a frozen state it did not follow that it was free from chemicals, since in the farms of Australia or South America, where the cattle and sheep were raised and where they were killed, there were no refrigerating appliances, and some preservative was needed to permit of the carcasses being wholesome when taken to the ship. For the most part the preservative used was the comparatively harmless one—borax or boracic acid. More powerful ones were also more dangerous. Dr. Hegner pleaded for the establishment of a Government department, where expert knowledge would be available and which would not forbid, but regulate, the use of chemical substances for preserving food.

Dr. Thresh, medical officer of health to the Essex County Council, who seconded the resolution, said that, though he had inquired of his fellow M.O.H.s, he had not heard of any cases of illness due to the use of preservatives in food. He pointed out, however, that, as the action of these substances was insidious, they might act injuriously without the mischief being set down to the real cause. Where there was any weakness of the organs of excretion the chemical substances ingested with food might not be wholly eliminated, and thus the seeds of disease be sown. Where such a risk existed it ought to be possible for people to know where they could get foods free from the addition of preservatives. Dr. Bell, who spoke next, seemed to think there was not much ground for Dr. Thresh's anxiety, and quoted cases in which borax had been consumed in considerable quantities without ill effects, and also pointed out that boracic acid was used of set purpose in cases where there was putrefaction of the urine. Dr. Simon, medical officer to the Local Government Board, renewed the demand for regulation, because at present it was difficult to say when it was right to prosecute. He thought that traders should state on the articles they sold what preservative had been used in the manufacture, and how much. As producers, middlemen, and retailers might all add a preservative, without knowing what the others put in, the consumer might get a triple dose of the drug, to the detriment of his health. This point was touched on also by Mr. Macmeikan, who is connected with margarine factories in Belgium and Holland. He pointed out that much of the fats used in this manufacture was imported from America, and had there been treated with preservatives to an extent the manufacturer could not know. He seemed to think that the manufacturers should not be held responsible for any excess of chemicals due to this cause. He, however, also desired to see a Government department established, and suggested that, among its other functions, it might investigate the new preservatives that were continually being brought out, and find out which were good.

Mr. F. W. Beck proposed the second resolution :—

"That, for the purposes of formulating the regulations mentioned in the foregoing resolution, a standing advisory committee should be formed, to consist of properly-qualified persons, with power to co-opt, for the purpose of determining the use of such preservatives."

In speaking to the resolution, Mr. Beck spoke of the present lack of finality in dealing with questions of adulteration, and contended that thereby the public was deprived of the protection it would have if the law were clearer. He quoted some cases which showed how unequal was the justice meted out in courts of summary jurisdiction in cases where there was an accusation of excessive use of preservatives, but agreed with the previous speakers that if a committee were appointed it should represent not only the officials but also the scientific and the commercial men who were interested in the subject. Both resolutions were carried unanimously, and one gentleman asked that they should be forwarded promptly to the President of the Local Government Board, as there was some prospect of a new Act dealing with the sale of food and drugs being passed shortly.

